# Granulomatous cutaneous T-cell lymphoma in a patient with atage IVB of mycosis fungoides: a case report and literature review

**DOI:** 10.3389/fonc.2026.1830889

**Published:** 2026-06-10

**Authors:** Zhu Lin, Jing Xue, Guozeng Ye, Shuang Li, Tingzhi Liu, Jiping Lang, Zhixin Zheng, Xiaoyan Li, Qinbo Wang, Junrong Chen

**Affiliations:** 1 Department of General Practice, The sixth Affiliated Hospital, Sun Yat-Sen University, Guangzhou, China; 2 Biomedical Innovation Center, The Sixth Affiliated Hospital, Sun Yat-sen University, Guangzhou, China; 3 Department of Pharmacy, The sixth Affiliated Hospital, Sun Yat-Sen University, Guangzhou, China; 4 Department of Hematology, The sixth Affiliated Hospital, Sun Yat-Sen University, Guangzhou, China; 5 Department of Dermatology, The sixth Affiliated Hospital, Sun Yat-Sen University, Guangzhou, China; 6 Department of Graceland Medical Center, The sixth Affiliated Hospital, Sun Yat-Sen University, Guangzhou, China

**Keywords:** CD4/CD8 double negative, cutaneous T-cell lymphoma, follicular helper T-cell, monoclonal, mycosis fungoides

## Abstract

**Background:**

Granulomatous mycosis fungoides (GMF) represents a distinct pathological variant of mycosis fungoides (MF), mycosis fungoides, originating from memory helper T cells, is the most prevalent form of primary cutaneous T-cell lymphoma. It is characterized as an inactive CD4+ non-Hodgkin lymphoma, where in some patients lesions gradually evolve into plaques or tumors over several years.

**Presentation:**

The patient presented with multiple masses on his trunk and limbs, featuring ulcers and scab formation, the patient was previously diagnosed with “psoriasis” before, however, clinicopathological correlation led to a diagnosis of granulomatous mycosis fungoides (GMF). The clinical and histological presentations of GMF are heterogeneous, and often mimic those of various inflammatory skin disorders. The patient’s erythema and mass were biopsied separately, and the tumor cell characteristics included positivity for CD3+ and negativity for CD7−, CD4−, and CD8−. Immunohistochemical evaluations showed diffuse positivity for both T-cell lineage markers (CD2 and CD3), EBV *in situ* hybridization showed EBER (−). Absence noted surrounding (CD56), indicating active hyperplastic states existing within the bone marrow composition. TCRβ and γ genes detection confirmed the presence of a clonal process.

**Conclusion:**

This report explores the clinical and histological manifestations of GMF, features distinguishing GMF from other granulomatous diseases, such as IGD, and the prognostic significance of distinguishing GMF from classic mycosis fungoides. We aimed to raise awareness among physicians regarding this rare disease and emphasize the characteristic histological features of GMF that can be confused with other types of mycosis fungoides.

## Introduction

Granulomatous mycosis fungoides (GMF) is a distinct histological variant of mycosis fungoides (MF). Classic MF originates from memory helper T-cells and is the most common primary cutaneous T-cell lymphoma (about 50% of cases of cutaneous lymphoma) (1) MF is an indolent CD4^+^ non-Hodgkin lymphoma in which skin lesions gradually evolve from patches to plaques and tumors over years. Approximately one-third of patients may experience lymph node and visceral involvement, along with pathological large-cell transformation in the advanced stages. The clinical and histological presentations of MF are heterogeneous and often mimic those of various inflammatory skin disorders (2, 3). Additional manifestations included urticarial or purpuric eruptions, papulovesicular lesions, nodular formations, erythematosquamous plaques, and ulcerated areas. Owing to this diverse array of dermatological features, cutaneous biopsies are frequently performed in patients with GMF to differentiate genuine GMF involvement from other inflammatory conditions. Furthermore, MF can be easily overlooked or misdiagnosed clinically because of its low tumor burden, atypical histopathological findings, and the absence of specific tumor cell markers during the early stages (4). Here, we present a case study of granulomatous cutaneous T-cell lymphoma and summarize its clinical characteristics, along with differential diagnoses and treatment options for T-cell lymphoma. Additionally, we discuss the clinical and histological manifestations unique to GMF, as well as the features that distinguish it from other granulomatous diseases, and explore the prognostic implications associated with differentiating GMF from classic MF.

## Case presentation

### Chief complaints

A 57-year-old male presented with multiple erythematous patches accompanied by scales across his body and lower limbs since April 2022, which varied in size from broad beans to palm-sized, without significant pruritus or systemic symptoms such as fever or weight loss. By March 2023, he had developed multiple masses on his trunk, both anteriorly and posteriorly, as well as on his limbs, featuring ulcers and scab formation. Initially, the patient was diagnosed with “psoriasis” at other medical facilities, where topical treatments proved ineffective after four courses of adalimumab led to symptom exacerbation; thus, treatment was discontinued. He reported a ten-year history of alcohol consumption averaging 100 grams per day but ceased drinking following disease onset while denying any familial predisposition towards similar conditions. He was admitted to the general practice department of the Sixth Affiliated Hospital of Sun Yat-Sen University on May, 2023.

### Physical examination

The examination revealed numerous erythematous lesions ranging in size from broad beans to palm-sized across the anterior trunk back limbs exhibiting bright red coloration accompanied by white flaky scales; certain areas displayed circular distribution patterns among trunk erythema. Multiple palpable skin masses were noted on both the abdomen and extremities, measuring dark red in color and clearly demarcated, presenting large black scabs without tenderness, yet demonstrating signs of infiltration. An enlarged lymph node measuring approximately 2 cm × 2 cm was palpated within the right axilla displaying hard consistency devoid adhesion surface skin lacking tenderness; another enlarged lymph node around three centimeters found that the right groin similarly firmly adhered to the surrounding tissue without discomfort, and the left groin exhibited a larger five-centimeter swollen gland and rigid attached adjacent dermal structures free of pain upon palpation. No remarkable abnormalities or evidence of joint inflammation was observed in the hair or nails.

### Investigations

#### Radiology

**Figure 1:**
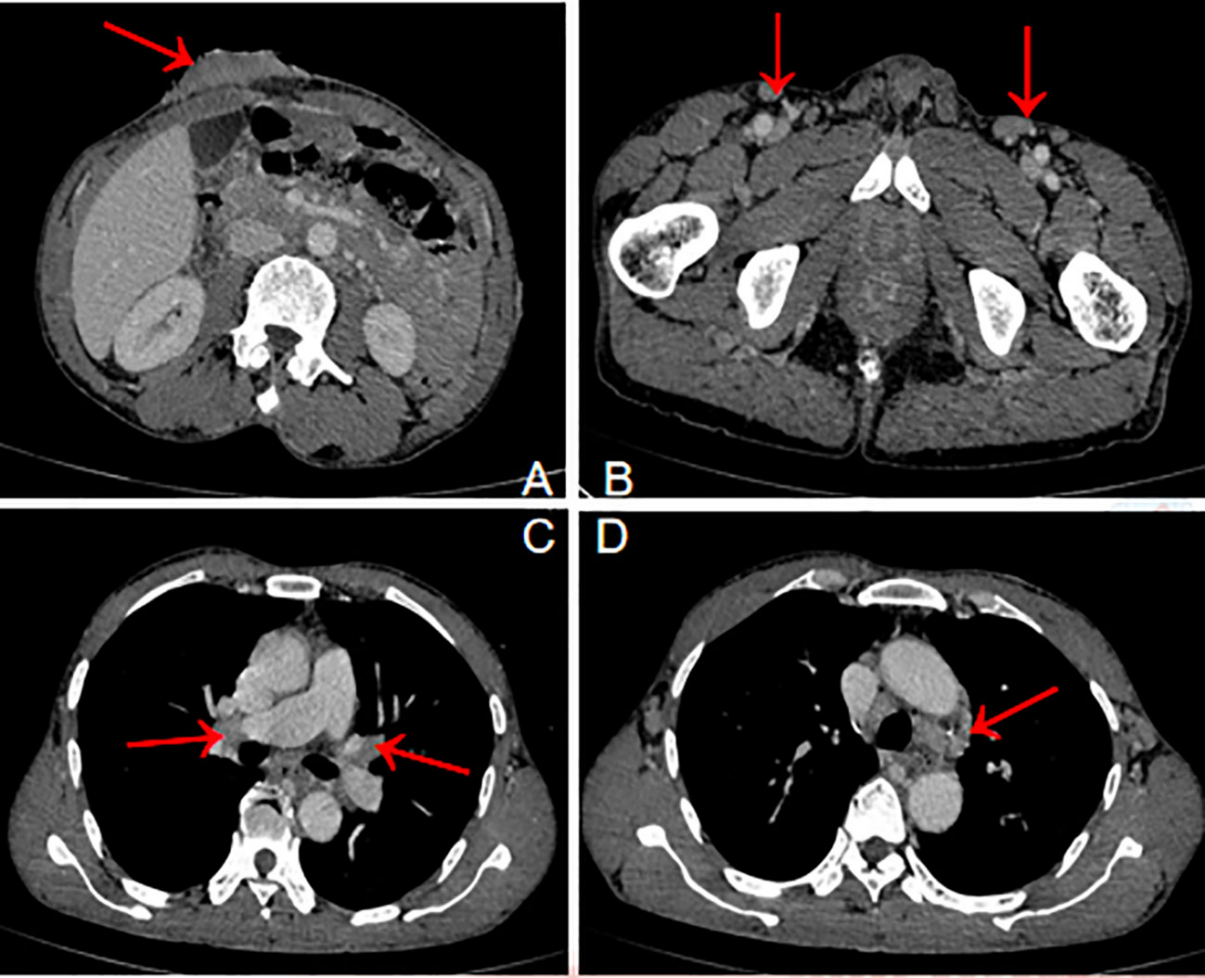
Irregular thickening of the skin near the papilla of the right middle and upper abdominal wall and the right chest wall was considered as a possible primary cutaneous T-cell lymphoma. Multiple enlarged lymph nodes in mediastinum, hilum of lung, retroperitoneum, bilateral supraclavicular fossa, axilla, paravascular iliac, and groin were considered as possible infiltration of lymphoma. Left renal vein and pancreatic parenchyma were changed under pressure. **(A)** abdominal skin masses, **(B)** swollen lymph nodes in the groin, **(C)** bilateral hilar enlargement of lymph nodes, **(D)** enlarged lymph nodes in mediastinum).

Enhanced CT scans encompassing the chest, abdomen, and pelvis indicated irregular thickening localized near papillae situated in the upper, middle abdominal wall, and right thoracic region, correlating with the patient’s prior medical history, suggesting a potential diagnosis of primary cutaneous T-cell lymphoma. Notably, multiple enlarged mediastinal, hilar retroperitoneal bilateral supraclavicular fossa axillary para-vascular iliac, and groin regions were identified, indicative of possible infiltration process-related malignancy affecting the renal vein and pancreatic parenchyma under pressure effects (**Figure 1**).

### Biochemical detection

#### Laboratory examinations

Laboratory Examinations were showed in **Table 1**, and results from serum immunofixed electrophoresis demonstrated monoclonal IgG-lambda- and GAM-lambda-type immunoglobulins characterized by oligoclonal bands. Prior to surgery, both liver and kidney functions remained within normal limits without significant abnormalities noted across the three thyroid function tests or tumor markers, including carbohydrate antigens CA125, CA15-3, CA19-9, carcinoembryonic antigen, and alpha-fetoprotein.

**Table 1:** The laboratory data of the patient.

Index	Result	Reference range
WBC	10.68×10^9^/L↑	3.5–9.5×10^9^/L
RBC	3.37×10^12^/L↓	4.3-5.8×10^12^/L
Hb	103.00 g/L↓	130–175 g/L
ALC	5.87×10^9^/L↑	0.8 - 4.0 × 10^9^/L
IgA	6.56 g/L↑	0.76 - 3.90 g/L
IgM	4.20 g/L↑	0.4 - 3.45 g/L
C3	1.38g/L	0.8 - 1.6 g/L
C4	0.05 g/L↓	0.1 - 0.4 g/L
β-1	4.00%↓	4.7% - 7.2%
β-2	7.30%↑	3.2% - 6.5%
ALB	27.75 g/L	40–55 g/L
TB	115.61 g/L↑	60–80 g/L
EBV-DNA	1.03×104 copies/mL	<500 copies/mL
IgG	61.09 g/L	7.0–16.0 g/L
γ-globulin	52.60%	10.0–20.0%
κ-FLC	10.84 g/L	3.0 - 19.0 mg/L
λ-FLC	16.82 g/L	6.0 - 26.0 mg/L

WBC, white blood cell; RBC, red blood cell; Hb, hemoglobin; ALC, absolute lymphocyte count; IgA, immunoglobulin A; IgM, immunoglobulin M; EBV-DNA, EBV-DNA quantification; IgG, immunoglobulin G.

Peripheral venous blood samples were collected from the patient. The absolute counts of peripheral blood CD3^+^, CD4^+^ and CD8^+^ T lymphocytes were determined by flow cytometry-based peripheral blood lymphocyte subset analysis. All assays were performed following the standard operating procedures, with instrument calibration and quality control conducted routinely to ensure detection accuracy. CD3 positive cells had an absolute count recorded as being greater than 3844/μl↑; CD4 + cells counted above 2064/μl↑, while CD8 + cells reached above 1564/μl↑.

#### Pathology

**Figure 2:**
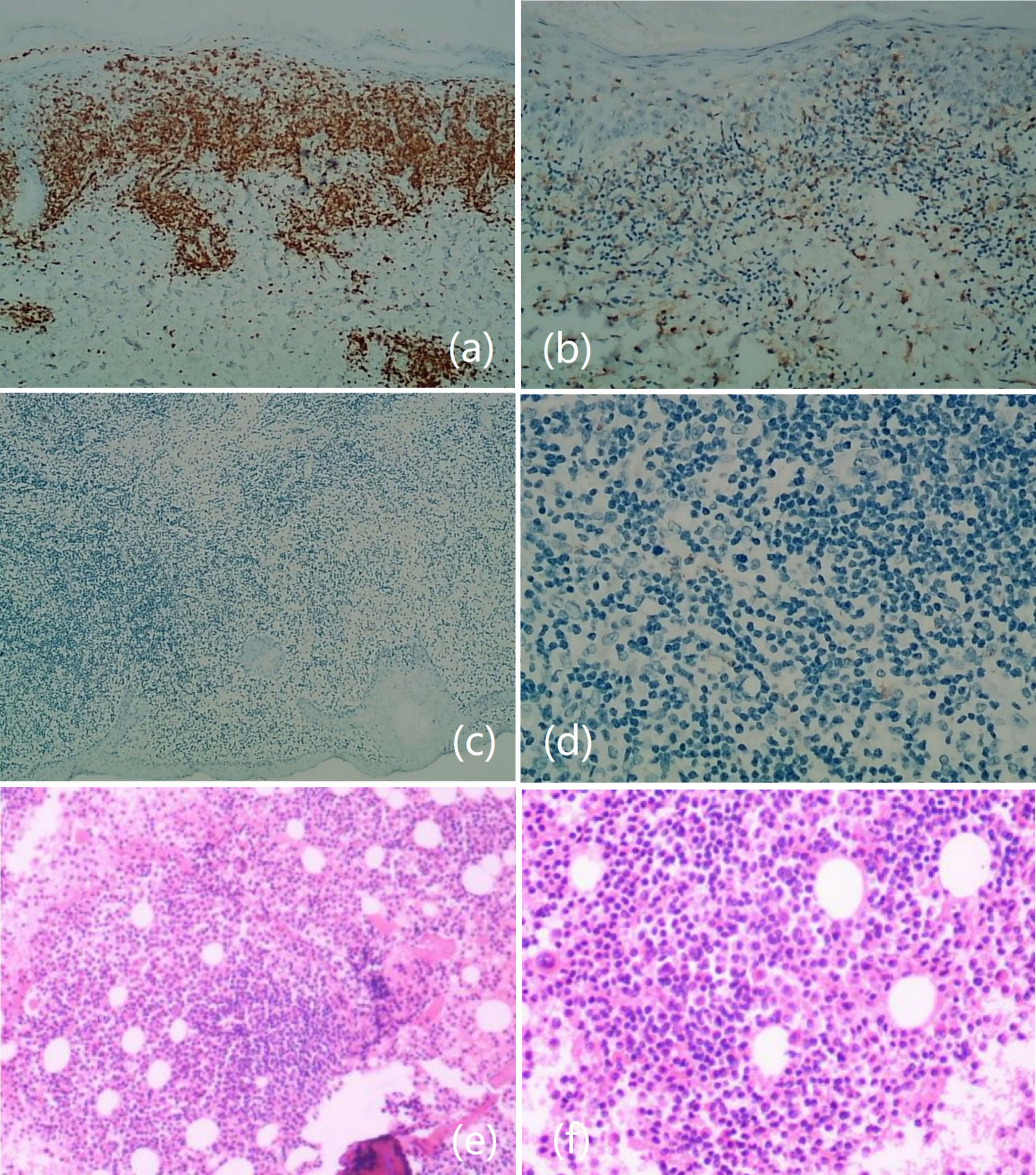
**(a)** showed the immunohistochemical staining of CD3 or CD2 in abdominal skin lesions. As pan‑T cell markers, CD3 and CD2 are usually co-expressed, and the tumor cells displayed diffuse and strong positive staining. **(b)** showed CD4 staining. The vast majority of tumor cells infiltrating the entire dermis were CD4-negative (counterstained in blue), while only a few residual reactive T cells showed focal CD4 positive expression (brown staining), which is consistent with the immunohistochemical conclusion of CD4 negativity. **(c, d)** showed EBER in situ hybridization with negative results. **(e, f)** demonstrated morphological changes of increased T-lymphocyte infiltration.

Breast mass biopsy results indicated skin tissue exhibiting local epidermal erosion along with epidermal thinning, with minimal migration patterns of lymphocytes into the epidermis; however, numerous infiltrative lymphoid cells were populated throughout the dermal layers, showing superficial zonation, while deep perivascular distributions presented nodular formations. During immunohistochemical assessment, T cell characteristics included positivity for CD3+ and negativity for CD7−, CD4−, and CD8−. PD1 exhibited scattered positivity (+), βF1 displayed strong positivity (+), partial weak expression was seen in some areas regarding CD20, and sparse + findings on CD56, whereas GRB showed focal + presence, indicating proliferation rates reflected by KI67 scoring around five percent overall, but peaking near 50 percent within localized hot zones (**Figure 2a**).

Chest erythema biopsy revealed after processing through deep wax masses, skin samples combined with slight subcutaneous fat tissues, illustrated fused keratosis within the basal layer of vacuolar degeneration, accompanied by increased invasion from atypical lymphoid populations invading epidermal structures, presenting band-like infiltration patterns among superficial dermal regions, alongside clustered infiltrations evident deeper in the dermis/subcutaneous adipose compartments. Immunohistochemical evaluations showed diffuse positivity for both tumor-associated markers (CD2 and CD3); however, only a partial weak expression of the marker (CD20) was detected. The remaining assessments involving other markers, such as (CD4), (CD8), (CD5), (CD7), (CD30) (PD1) and EMA, yielded negative results (**Figure 2b**).

Epstein-Barr virus (EBV) *in situ* hybridization revealed EBER (−). T-cell clonality evaluation confirmed the detection of monoclonal rearrangements pertaining to the TCRβ and γ genes, which led to the diagnosis of cutaneous T-cell lymphoma, correlated clinical presentations associated with skin rash morphology, and comprehensive analyses derived from both histopathological/immunophenotypic data. suggesting granuloma fungoides classification reflecting transitional stages identified through phenotypes displaying features like (CD20+, CD4-/CD8-and cytotoxic+) variations (**Figures 2c, d**).

Bone marrow smear analysis revealed part-positive findings related specifically towards marker(CD2); large clusters indicative of underlining presence marked positively concerning(CD3); less frequent scatterings observed linked back towards marker(CD20); absence noted surrounding(CD56), indicating active hyperplastic states existing within the bone marrow composition wherein proportions attributed directly towards respective cellular types accounted upwards nearing twenty-three percentage points whilst plasma cell ratios and elevated beyond ten-point six percentages, respectively. CD3+CD5-mature t-cells constituted nearly seventeen point five percentages relative to the total nuclear population, showing considerable deviations tied back towards their corresponding phenotypic profiles (**Figures 2e, f**).

### Final diagnosis

Granulomatous cutaneous T-cell lymphoma (Stage IVB of Mycosis fungoides); monoclonal immune globulinemia.

### Treatment

**Figure 3:**
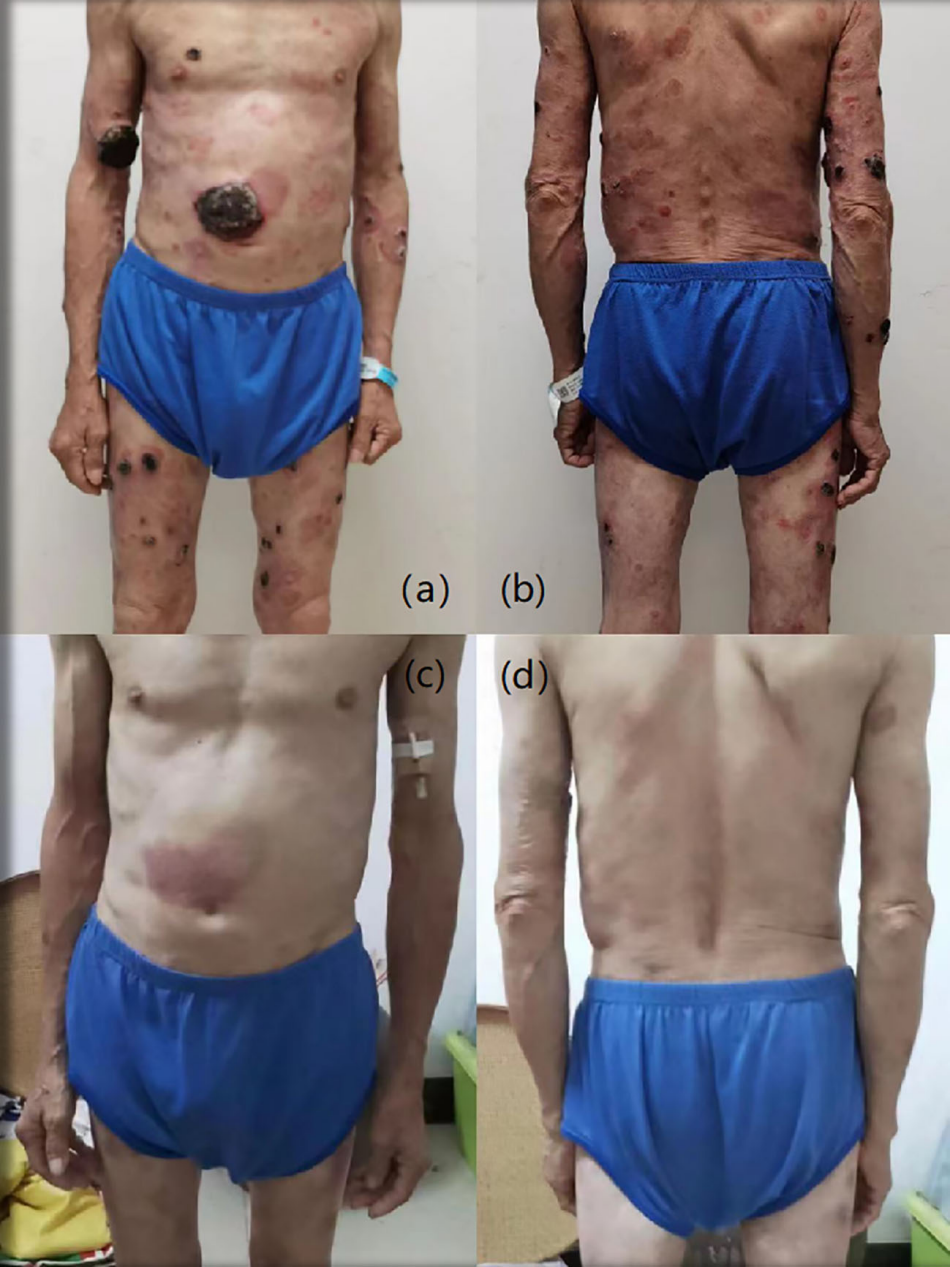
The images of patient before and after the treatment. **(a, b)** Multiple erythema can be seen in front, back and limbs of the trunk, the size of broad beans to the palm, bright red color, accompanied by white flake scales, ring distribution of erythema in part of the trunk, multiple skin masses and ulcers can be seen in the abdomen and limbs. **(c, d)** The skin lesions of the patient were significantly improved, the masses disappeared in front and back of the trunk and limbs.

Based on the patient’s condition, an individualized treatment plan was developed that included interferon therapy (recombinant human interferon alpha-2B for injection at a dosage of 3 million IU per dose via intramuscular injection every other day), methotrexate tablets administered orally at a dosage of 15 mg once weekly, and acitretin as part of the oral chemotherapy regimen. Fluconazole tablets were administered orally at a dose of 100 mg once daily. Additionally, a combination targeted chemotherapy comprising ‘Cedaaniline + vibutuximab + cyclophosphamide + pirarubicin + vindesine + etoposide + dexamethasone’ was employed. Following this treatment protocol, rapid improvement in the lesions was observed, with plaques and nodules flattened within one month. After two months of therapy, all plaques and nodules resolved completely (**Figure 3**).

## Results of the literature review

**Figure 4:**
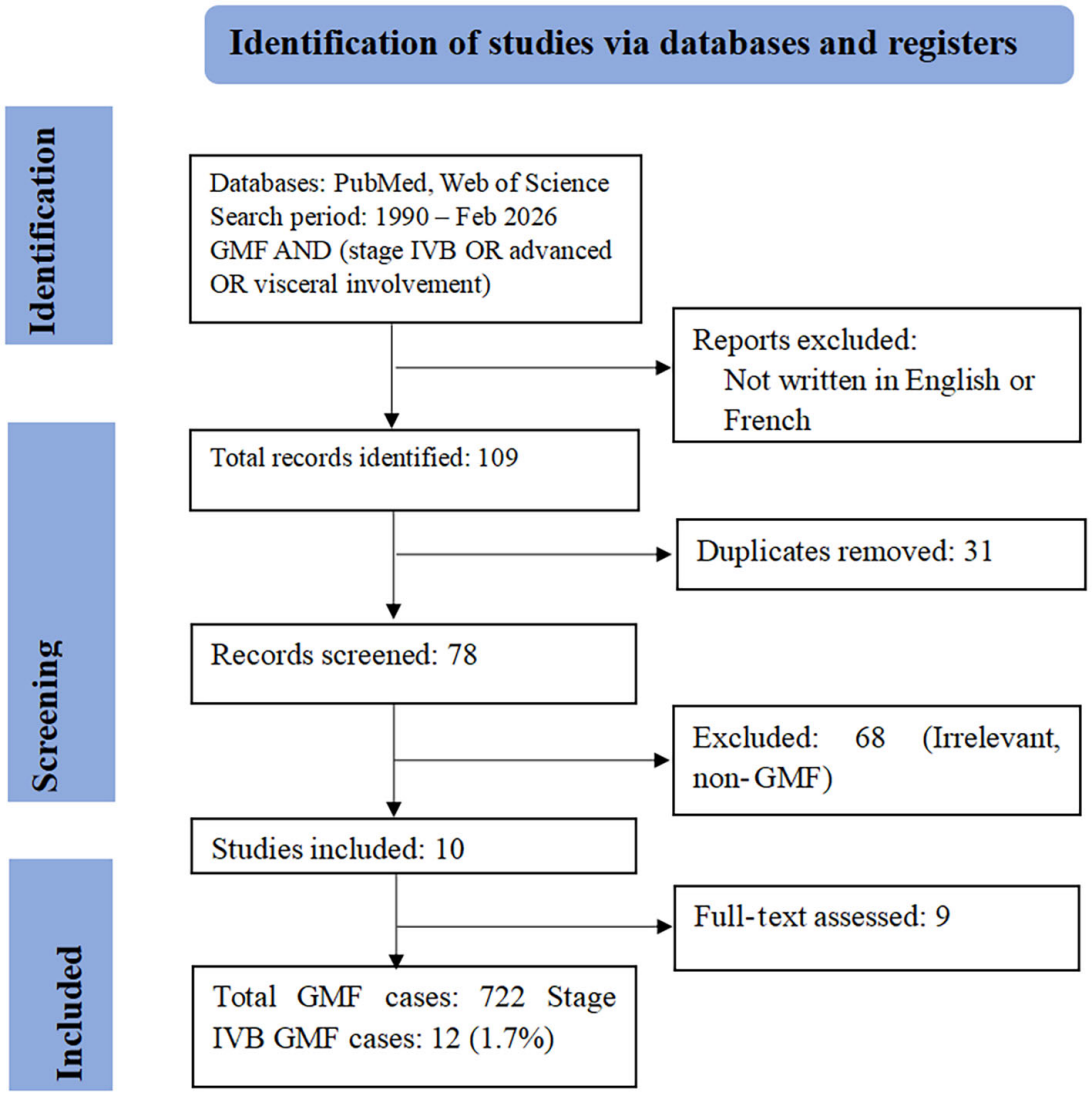
Flow diagram of the process for narrative review.

In order to better understand and contextualize this case report, observations with cases and data reported in the international literature were compared. A systematic literature search was performed in PubMed and Web of Science from 1990 to February 2026, using keywords related to granulomatous mycosis fungoides, stage IVB, advanced disease, and visceral involvement. A total of 48 eligible studies, including case reports, case series, and literature reviews published in English or Chinese, were enrolled for qualitative synthesis (**Table 2**; **Figure 4**). These studies comprised 116 cases of granulomatous mycosis fungoides, among which 7 patients (6.0%) were diagnosed at stage IVB, characterized by extensive skin tumors or erythroderma, lymph node involvement, and distant visceral or bone marrow metastasis (5–11). Compared with classic mycosis fungoides, the granulomatous variant exhibited more aggressive clinical behavior, higher rates of B symptoms, and relatively poor responses to skin directed therapies. Systemic chemotherapy, targeted agents such as brentuximab vedotin, and local or total skin radiotherapy represented the main therapeutic approaches, with a median overall survival of approximately 28 months in stage IVB patients. Collectively, these findings highlight the rarity, aggressive phenotype, and challenging clinical management of advanced granulomatous variants of mycosis fungoides.

**Table 2:** Summary of key literature data (granulomatous mycosis fungoides, IVB stage).

Reference	Year	Total cases	IVB stage cases	Clinical characteristics	Immunophenotype / Pathology	Treatment	Prognosis
Motamedi M, et al. (5)	2024	116	7	Median age 64 years; distal extremities lesions; B symptoms (85.7%); lung, liver, bone marrow involvement	CD4+CD8− (6/7); TCR rearrangement (100%); granulomatous infiltrate with atypical T cells	Chemotherapy (CHOP/ICE) + Brentuximab vedotin + radiotherapy	Median OS 28 months; 3 deaths (42.9%)
Fischer M, et al. (6)	2021	2	0	Case 1: 52-year-old, diffuse livid plaques all over the body.Case 2: 88-year-old male, plaques and ulcerative tumours on trunk and head.No specific clinical features for granulomatous MF.	Malignant T-lymphocyte infiltration, monoclonal TCR-gamma rearrangement, granulomas and multinucleated giant cells.	Case 1: Multiple chemotherapy regimens with progression.Case 2: Local PUVA plus electron radiotherapy.	Granuloma formation has no definite prognostic value; disease course varies from aggressive to long-term stable.
Jung JM, et al. (7)	2021	571	5 cases of stage IV (1.0%)	Mean age at diagnosis: 12.2 years; onset age: 8.6 years. Male predominance. Main subtypes: hypopigmented MF, classic MF. Most patients at stage I.	CD4+ (49.5%), CD8+ (39.5%)	Narrowband UV-B was the most common therapy (35.2%).	66.3% alive with disease, 29.8% complete remission, 3.9% dead. Granulomatous MF and stage II correlate with poor prognosis.
Miyashiro D, et al. (8)	2022	727 (673 MF, 54 SS)	Extracutaneous involvement was rare	Median age: 51.8 years; older in erythrodermic MF (60.2) and SS (60.9). Male 51.2%, female 48.8%. Main subtypes: classic MF, folliculotropic MF, granulomatous slack skin, erythrodermic MF, hypopigmented MF and poikilodermatous MF. Higher proportion of hypopigmented MF than previous reports.	Related histopathological and immunopathological data analyzed; no detailed immunophenotype statistics listed.	Treatment details not stated in the abstract.	Early-stage: 5/10/20/30-year OS: 97.3%, 92.4%, 82.6%, 82.6%.Advanced-stage: 5/10/20/30-year OS: 58.6%, 42.7%, 20.8%, 15.4%.Poor prognostic factors: SS, folliculotropic MF, erythrodermic MF, advanced stage, age ≥60 years, elevated LDH.
Ishida M, et al. (9)	2010	1	No relevant data	75-year-old Japanese female. Presented with brownish maculae on trunk and extremities. Lesions relapsed and expanded after temporary improvement.	Neoplastic cells: CD3+, CD8+, betaF1+, partial TIA-1+. CD8-positive GMF is extremely rare.	Initial PUVA therapy relieved most rashes, but the disease relapsed later.	Prognosis of CD8-positive GMF remains controversial; further research is needed.
Li JY, et al. (10)	2013	27	No relevant data	Skin manifestations similar between GMF and classic MF.	Atypical lichenoid infiltrate: CD4+ CD8− lymphocytes, interstitial histiocytes and perivascular granulomas with giant cells.	GMF had lower response rates: topical therapy (57% vs 83%); ultraviolet therapy (62% vs 90%).	GMF had lower 5-year (59%) and 10-year (33%) PFS than classic MF (84%, 56%).
Patil S, et al. (11)	2025	1	No relevant data	82-year-old male, 6-month itchy lesions on scalp, face, limbs and trunk. Multiple erythematous firm nodules with ulceration. Complicated with poorly controlled diabetes.	Tumor cells: CD3+, CD4+, CD8+, CD20-. GMF total reported cases < 100; majority are CD4-positive, CD8-positive GMF is extremely rare.	Prior local treatment showed no improvement; no specific regimen mentioned in abstract.	CD8-positive GMF tends to affect elderly females generally; this case has no adverse prognosis.
Kogut M, et al. (12)	2015	1	Subtype of MF	44-year-old; presented with painful ulcers on his left ear, with purulent fistulas and tender ulcerations.	Ppidermotropism of CD4-positive lymphocytes by only scattered CD8-positive T cells	Low-dose interferon alfa (INF-α)+photochemotherapy	Complete remission of the ear tumor, not found any signs of systemic involvement.
Nakagawa Y, et al. (13)	2021	1	No relevant data	63-year-old female, Annular erythematous plaques with coalescent red papules (ring-like distribution) on hands, elbows, legs and buttocks;	CD3dim+CD4+CD7−CD25+CCR4+ phenotype; Ki-67 proliferation index was 39.5%.	Re-PUVA regimen combined with oral etretinate	Combined therapy clear the granulomatous skin lesions, control for nearly 2 years
Rojansky R, et al. (14)	2020	2	Unspecified	Case 1: 38-year-old male, 19-year history, presenting with papulosquamous, granulomatous, verrucous lesions, and progressively aggravated ulcerated plaques and tumors. Case 2: 69-year-old male, 13-year history of progressive diffuse cutaneous lesions, characterized by hypertrophic and eroded plaques.	Atypical CD4+ T-cell infiltration in skin lesions;	Unspecified	Unspecified

OS, overall survival; PFS, progression-free survival; PR, partial response; CR, complete response; TSEB, total skin electron beam therapy; CHOP, cyclophosphamide, doxorubicin, vincristine, prednisone; ICE, ifosfamide, carboplatin, etoposide.

## Discussion

Granulomatous mycosis fungoides (GMF) is recognized as a very rare histological variant within mycosis fungoides (MF). First described by Ackerman and Flaxman in 1970, GMF displays typical pathological features associated with MF but lacks distinct clinical manifestations. It may present as papules, plaques, nodules, or ulcers, along with erythroderma and heterochromic lesions (12). Furthermore, GMF can affect non-cutaneous organs, including the lymph nodes, liver, spleen, small intestine, central nervous system, and peripheral blood (15). Due to its nonspecific clinical presentation, diagnosis is frequently delayed by several years; retrospective analysis has revealed that the average time interval from symptom onset to GMF diagnosis spans approximately 8.4 years (16). Diagnosis primarily relies on pathological examination revealing various histopathological changes, such as scattered histiocytic cells, giant cells, sarcoid-like granulomas, cricogranulomatous granulomas, or nodular granulomas, each of which is prevalent in tissue specimens from single sites, whereas multiple forms may be identified across different locations (17). Li et al. (16) noted that granulomas featuring multinucleated giant cells accompanied by eosinophils are predominantly found in stromal perivascular regions, where they often exhibit pronounced central areas rich in histiocytes, resembling a cricogranulomatous pathology.

The histiocytic component of granulomatous mycosis fungoides (GMF) constitutes at least 25% and may be confused with or overlapping the large cell transformation seen in mycosis fungoides (MF), which can be distinguished through immunohistochemical staining for CD68 and CD163 (18). Differential diagnoses based on histopathological examination include interstitial annular granuloma, granulomatous drug reactions, cutaneous Rosai-Dorfman disease, and peripheral T-cell lymphomas such as angioimmunoblastic T-cell lymphoma (AITL). Thorough medical history is essential to exclude potential medical causes. If follicular helper T-cell markers (PD-1, CXCL-13, Bcl-6) are positive in B-cell clusters with abnormal EBER *in situ* hybridization, AITL should be highly suspected (19). S100 protein detection and auxiliary examinations help exclude Rosai-Dorfman disease, and multiple targeted biopsies facilitate the diagnosis of interstitial granuloma annulare. In addition, it must also be differentiated from granulomatous cutis laxa characterized by more pronounced histological features, including diffuse histiocytic infiltration accompanied by giant cells and loss of elastic fibers, clinically presenting as skin fold atrophy and sagging particularly noted around the axillary region and groin. The diagnosis of GMF poses challenges, especially when epidermotropism is not readily apparent and extensive granulomatous lesions obscure neoplastic lymphocyte infiltration. In such instances, multipoint sampling coupled with special staining techniques, immunohistochemistry assessments, and T-cell receptor gene rearrangement analysis may facilitate differential diagnosis (13). Sequencing of the T-cell receptor (TCR) gene serves as a critical diagnostic criterion for GMF (14). An important distinguishing feature shared between GMF and Cutaneous T-cell lymphoma (CTCL) lies within the clonality observed in studies involving TCR rearrangements (20), and Th1 and Th2 cells have been implicated in inducing granulomas in various diseases, including lymphoproliferative disorders (21). Prior investigations indicate that the formation of these granulomas relies heavily on CD4+T cells transitioning from an initial Th1 response towards sustained dominance by Th2 responses (22). *In vitro* analyses have demonstrated that a shift towards Th2 phenotypes alongside increased production of interleukin-4 or interleukin-13 promotes granulation tissue formation across other pathological contexts (23). Shimauchi etal. (24) reported non-polarized T cell phenotypes (CXCR3+ and CCR4+) in skin biopsy specimens from patients diagnosed with GMF, suggesting that the depolarizing effects on Th2 populations correlate significantly with granulation tissue development. The literature indicates that MF exhibits a granulomatous reaction post-treatment using agents such as bexarotene or IFN-γ, suggesting possible drug-induced mechanisms underlying this phenomenon (25, 26).

Next-generation sequencing (NGS) has emerged as a cutting-edge tool for dissecting the molecular landscape and identifying driver gene alterations in advanced cutaneous T-cell lymphoma (CTCL) (27). Recent genomic studies have revealed recurrent mutations in epigenetic regulators (TET2, DNMT3A), JAK-STAT signaling (JAK3, STAT5B), tumor suppressors (TP53, CDKN2A), and T-cell activation/differentiation genes (CCR4, RHOA, PLCG1) across aggressive CTCL variants (28). Notably, these mutations are strongly associated with tumor progression, large cell transformation, drug resistance, and inferior survival in advanced stage disease. Our patient presented with Stage IVB granulomatous mycosis fungoides (GMF) exhibiting an extremely rare double negative (CD4-/CD8) immunophenotype, a distinct clinicopathological correlation linked to more aggressive clinical behavior (29, 30). This case showed a rare CD4−/CD8 double negative phenotype, which is a rare clinicopathological correlation in GMF. No *in vitro* evidence supports that this phenotype directly causes tumor invasion. Given the highly aggressive nature and unique immunophenotype of this GMF variant, we hypothesize that it may harbor specific, non-random driver gene mutations or pathway disruptions distinct from classic CD4+MF. Potential candidate alterations include epigenetic dysregulation via TET2/DNMT3A, aberrant JAK-STAT activation, or cytotoxic program related gene mutations, which could underlie the loss of CD4/CD8 expression and heightened invasiveness. Further studies employing deep NGS in larger cohorts of double-negative GMF are warranted to define its mutational signature, clarify molecular mechanisms, and identify actionable therapeutic targets.

Therapeutic approaches for managing GMF align closely with those employed for classical MF (31), incorporating glucocorticoids alongside interferon therapy, while considering psoralen long-wave ultraviolet light treatment (PUVA), potentially augmented by radiotherapy or chemotherapy. Notably, extrandermal manifestations associated with GMF tend to occur earlier than those observed in classical MF cases, and their responsiveness to localized treatments, including ultraviolet exposure, appears diminished relative to classical MF. Accelerated progression rates often lead to poorer prognosis pre-treatment outcomes documented historically, wherein initially perceived favorable prognostic indicators linked to granular reactions were later contradicted, which revealed an approximately 45% incidence among highly malignant tumors, exhibiting similar reactions correlating negatively with survival outcomes, frequently attributed to tumor advancement compounded by secondary infections (32).

Studies addressing therapeutic interventions specific to GMF remain sparse, and some researchers demonstrate detailed management strategies applied during hospitalization involving PUVA (2–5 J/cm² thrice weekly over 5 weeks totaling 61·5 J/cm²), abtretinate administration (20 mg/daily), supplemented concurrently with IFN γ (1×106 IU administered three times weekly) (33–35). Nevertheless, the prominent therapeutic efficacy demonstrated in this case is based on limited experience from a single patient and cannot be extrapolated to establish a standardized treatment strategy for all patients with GMF. Caution should be taken when applying this combined regimen in clinical practice until validated by larger scale clinical studies.

## Data Availability

The original contributions presented in the study are included in the article/supplementary material. Further inquiries can be directed to the corresponding authors.
